# Development of reverse transcription loop-mediated isothermal amplification assays for point-of-care testing of avian influenza virus subtype H5 and H9

**DOI:** 10.5808/GI.2020.18.4.e40

**Published:** 2020-12-14

**Authors:** Songzi Zhang, Juyoun Shin, Sun Shin, Yeun-Jun Chung

**Affiliations:** 1 Department of Biomedicine & Health Sciences, Graduate School, The Catholic University of Korea, Seoul 06591, Korea; 2 Department of Microbiology, College of Medicine, The Catholic University of Korea, Seoul 06591, Korea; 3 Precision Medicine Research Center, College of Medicine, The Catholic University of Korea, Seoul 06591, Korea; 4 Integrated Research Center for Genome Polymorphism, College of Medicine, The Catholic University of Korea, Seoul 06591, Korea; 5 ConnectaGen, Hanam 12918, Korea

**Keywords:** avian influenza virus, H5 subtype, H9 subtype, RT-LAMP

## Abstract

Avian influenza (AIV) outbreaks can induce fatal human pulmonary infections in addition to economic losses to the poultry industry. In this study, we aimed to develop a rapid and sensitive point-of-care AIV test using loop-mediated isothermal amplification (LAMP) technology. We designed three sets of reverse transcription LAMP (RT-LAMP) primers targeting the matrix (M) and hemagglutinin (HA) genes of the H5 and H9 subtypes. RT-LAMP targeting the universal M gene was designed to screen for the presence of AIV and RT-LAMP assays targeting H5-HA and H9-HA were designed to discriminate between the H5 and H9 subtypes. All three RT-LAMP assays showed specific amplification results without nonspecific reactions. In terms of sensitivity, the detection limits of our RT-LAMP assays were 100 to 1,000 RNA copies per reaction, which were 10 times more sensitive than the detection limits of the reference reverse‒transcription polymerase chain reaction (RT-PCR) (1,000 to 10,000 RNA copies per reaction). The reaction time of our RT-LAMP assays was less than 30 min, which was approximately four times quicker than that of conventional RT-PCR. Altogether, these assays successfully detected the existence of AIV and discriminated between the H5 or H9 subtypes with higher sensitivity and less time than the conventional RT-PCR assay.

## Introduction

Influenza A viruses, which are classified into 18 hemagglutinin (HA) and 11 neuraminidase subtypes based on their antigenic properties, have been found to infect not only avian species, but also multiple mammalian hosts including humans. In particular, avian influenza viruses (AIVs) belong to the category of influenza A viruses, which induce human infections and cause economic losses annually [1-3].

Starting in late 2003, simultaneous outbreaks of highly pathogenic avian influenza virus (HPAIV) in poultry occurred across diverse Asian countries, mainly caused by influenza H5N1 virus [2,3]. The outbreaks spread widely throughout the globe [4], and caused several human infections, including fatal cases [5]. In addition to HPAIVs, low-pathogenic avian influenza viruses (LPAIVs), which usually produce mild or no symptoms in birds, can induce pathogenic avian influenza by antigenic drift or shift, and can also induce human infection [2]. Indeed, the H9 subtype AIV, an LPAIV, was reported to be capable of human infection [6], and the World Health Organization warned that the H9N2 subtype could trigger a global influenza outbreak in humans, albeit with relatively lower pathogenicity than H5N1 or H7N9. Therefore, identification of HPAIVs and LPAIVs would be important to control avian influenza and potential human pandemic infections.

To identify AIVs at the early stage of disease, a rapid, specific, and sensitive detection method is required. Polymerase chain reaction (PCR) and reverse transcription PCR (RT-PCR) are the most commonly used tools, serving as a gold standard for the diagnosis of viral infections including influenza. However, these methods require specialized equipment and trained personnel for the entire experimental process [7], which makes these assays hard to use for point-of-care testing (POCT) in the field. In addition, ordinary PCR or RT-PCR requires about 2 hours to process and well-purified nucleic acids because the reaction is sensitive to PCR inhibitors.

Notomi et al. [8] developed a new nucleic acid amplification technology, loop-mediated isothermal amplification (LAMP), which can amplify target nucleic acids at a consistent temperature without changing the temperature for denaturation, annealing, and extension. LAMP only needs strand displacement by using the Bst DNA polymerase enzyme for nucleic acid amplification, so it can be amplified under isothermal conditions with a high amplification efficiency [8]. LAMP technology has a number of advantages for POCT [9-11]. For example, the time required for LAMP assays is much shorter than that for PCR, and LAMP assays can detect the target nucleic acid with high sensitivity without the equipment needed for thermal cycling. Furthermore, diverse detection methods can be applied, such as turbidity, colorimetric detection, and fluorescent dye incorporation.

Due to those advantages, LAMP technology has been widely applied to detect various pathogens and its applications have been advanced to include several different forms of assays, such as reverse transcription LAMP (RT-LAMP) and multiplex LAMP [12,13]. In particular, RT-LAMP assays have been found to be an efficient tool to detect RNA viruses such as the severe acute respiratory syndrome coronavirus 2, influenza, dengue, and West Nile viruses [14-22]. In RT-LAMP, reverse transcription of viral RNA and cDNA amplification can be processed in a single tube as one step under isothermal conditions. The straightforwardness of RT-LAMP is ideal for on-site field testing or diagnostics in a POCT setting where laboratory equipment is limited [13]. Together, RT-LAMP can be a simple and effective tool to detect and distinguish diverse AIV subtypes in the poultry field or at clinical quick-check desks [19].

In this study, we developed RT-LAMP assays that can detect a universal target for AIV and can discriminate between the H5 and H9 subtypes of AIV. We validated the sensitivity, specificity, and processing time of our assay.

## Methods

### Primer design for RT-LAMP assays

The nucleotide sequences of the matrix (M) gene and the HA genes from the H5 and H9 subtypes (H5-HA and H9-HA, respectively) were retrieved from the Influenza Research Database (IRD) and Global Initiative on Sharing All Influenza Data (GISAID), and aligned using DNAStar (Lasergene, Madison, WI, USA). The RT-LAMP assay primers were designed based on M, H5-HA and H9-HA sequence alignments by using the software PrimerExplorer version 5 (http://primerexplorer.jp/lampv5e/). The feasibility of all sets of primers was then subsequently validated using the BLAST program.

### Preparation of target RNAs

Full-length fragments of the AIV M, H5-HA, and H9-HA genes with the T7 promoter sequence (TAATACGACTCACTATAGGGAGA) were chemically synthesized and cloned into a pTwist Amp High Copy plasmid (Twist Bioscience, South San Francisco, CA, USA). The RNA was transcribed by the universal M13F and M13R-pUC primers using the T7 RiboMAX Express Large Scale RNA Production System (Promega, Madison, WI, USA). The transcribed RNA was 10-fold serially diluted from 10^5^ copies/μL to 10 copies/μL, and used as a template for RT-LAMP and RT-PCR.

### RT-LAMP

The RT-LAMP reaction was carried out as described elsewhere [18-20]. In total, 25 μL of a mixture containing 0.2 μM of each outer primer (F3 and B3), 1.6 μM of each inner primer (FIP and BIP), 0.8 μM of each loop primer (LF and LB), 8 U of the Bst 2.0 DNA polymerase (New England Biolabs, Hitchin, UK), 2 U of AMV reverse transcriptase (New England Biolabs), 8 mM of MgSO_4_ (New England Biolabs), 1.4 mM of each dNTPs (Thermo Fisher Scientific, Waltham, MA, USA), 1× isothermal amplification buffer (New England Biolabs) and 0.4 M N-methylformamide (NMF) and isobutylamide (IBA) was prepared. For the real-time assay, 1 U of SYTO9 stain (Thermo Fisher Scientific) was added. One microliter of transcribed RNA was added to the respective tube. The reaction was carried out at 60°C for the H5-HA gene or at 68°C for the M gene and the H9-HA gene in a thermal cycler or CFX96 Touch Real-Time PCR Detection System (Bio-Rad Laboratories, Hercules, CA, USA). The reaction was terminated by heating at 80°C for 10 min. The fluorescence curve was captured in real time, and the result was illustrated as a graph on the monitor of the real-time system, verifying the amplification. RT-LAMP products were then evaluated by electrophoresis using 1.5% agarose gels to ensure the products of amplification reaction.

### RT-PCR

One-step RT-PCR was performed with the AIV Multi-tube RT-PCR Kit (iNtRON Biotechnology, Seongnam, Korea), which was approved by the Animal and Plant Quarantine Agency of Republic of Korea, according to the manufacturer’s instructions. The reaction cycling conditions were as follows: 30 min of RT at 45°C, RT inactivation and polymerase activation for 5 min at 94°C, 40 cycles at 94°C for 30 s, 55°C for 60 s and 72°C for 60 s, and final extension for 5 min at 72 °C. The RT-PCR products were then evaluated by electrophoresis using 1.5% agarose gel. The expected product sizes were 378 bp for the M gene, 311 bp for the H5-HA gene, and 252 bp for the H9-HA gene, respectively.

## Results

### Primer design and optimization of RT-LAMP assays

**Fig. 1.**
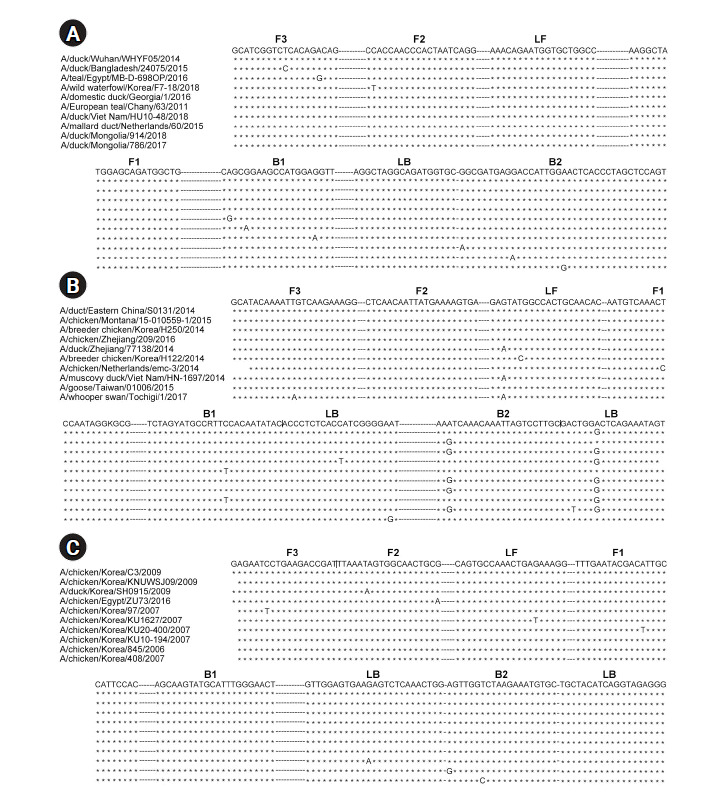
Primer design for the reverse transcription loop-mediated isothermal amplification assays. Multiple sequence alignments of the M (A), H5-specific (B), and H9-specific (C) HA genes with 10 nucleotide sequences from BLAST. The F3, F2, LF, F1, B1, LB, B2, and B3 regions are indicated above the sequences. M, matrix; HA, hemagglutinin.

**Fig. 2.**
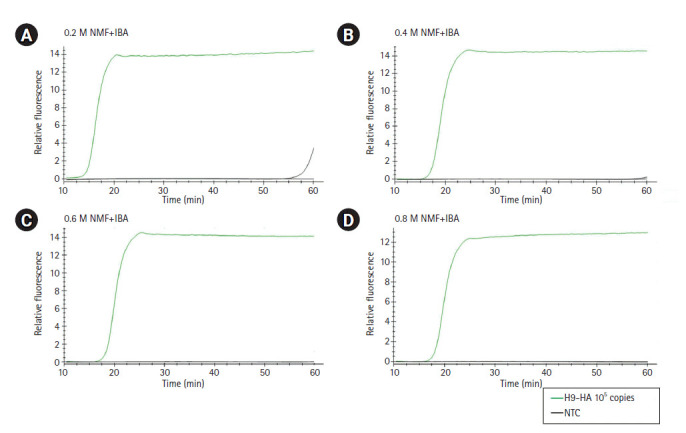
Optimization of the combinations of additives (NMF and IBA) in the reverse transcription loop-mediated isothermal amplification reaction mixture. (A) 0.2 M NMF + IBA. (B) 0.4 M NMF + IBA. (C) 0.6 M NMF + IBA. (D) 0.8 M NMF + IBA. The x-axis represents the time for RT-LAMP reaction; the y-axis represents the relative fluorescence signal. NMF, N-methylformamide; IBA, isobutylamide; NTC, negative control; RT-LAMP, reverse transcription loop-mediated isothermal amplification.

For RT-LAMP, primers were designed for the M, H5-HA, and H9-HA genes. The six primer sets for each target gene comprised two outer primers (F3 and B3), two inner primers (FIP and BIP), and two loop primers (LF and LB). In this study, *in vitro*–transcribed full-length RNA fragments of the M, H5-HA, and H9-HA genes were used as templates. Two to three different primer sets for each gene were tested and the sets that demonstrated the best amplification performance were selected for further analysis (Supplementary Fig. 1). The sequence information of the final RT-LAMP primer sets is available in Table 1. All selected primers in this study were designed within a conserved region of AIVs isolated from diverse poultry (Fig. 1). To minimize nonspecific amplification, we tested four different combinations of NMF and IBA in the reaction mixture (0.2 M NMF + IBA, 0.4 M NMF + IBA, 0.6 M NMF + IBA, 0.8 M NMF + IBA) using the H9-HA RT-LAMP set. When we applied a specific target template (H9-HA), all four reaction conditions showed significant amplification signals within 20 min regardless of the combinations of the additives (Fig. 2). When we applied a negative control template, the reaction mixtures with 0.4, 0.6, or 0.8 M NMF + IBA did not show any nonspecific signals during the whole process (60 min); however, the reaction containing 0.2 M NMF + IBA showed a subtle nonspecific signal after 50 min (Fig. 2). Therefore, we decided to add 0.4 M NMF + IBA for all three RT-LAMP reactions.

### Specificity of the RT-LAMP assays

**Fig. 3.**
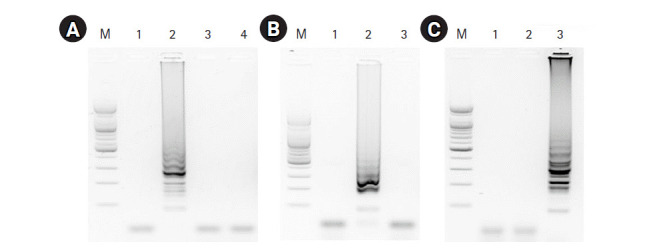
Specificity of the RT-LAMP assays. (A) The RT-LAMP products of the RT-LAMP assay for M gene were electrophoresed with 1.5% agarose gel: lane 1, negative control; lane 2, synthesized M RNA template; lane 3, synthesized H5-HA RNA template; lane 4 synthesized H9-HA RNA template; lane M, 100-bp DNA marker. (B, C) RT-LAMP products for H5-specific and H9-specific HA genes were electrophoresed with 1.5% agarose gel, respectively: lane 1, negative control; lane 2, synthesized H5-HA RNA template; lane 3, synthesized H9-HA RNA template; lane M, 100-bp DNA marker. RT-LAMP, reverse transcription loop-mediated isothermal amplification; M, matrix; HA, hemagglutinin.

To evaluate the specificity of the three RT-LAMP assays, we applied matched and non-matched template RNA samples including a negative control (no template). The RT-LAMP assay for the universal M target showed an amplification product with the M RNA template, but no amplification product was detected with for the HA RNA templates (H5 and H9) or the negative control template (Fig. 3A). The RT-LAMP assays for H5-HA and H9-HA showed target-specific amplification products without any cross-reaction with non-matched templates or the negative control (Fig. 3B and 3C).

### Sensitivity of the RT-LAMP assays

**Fig. 4.**
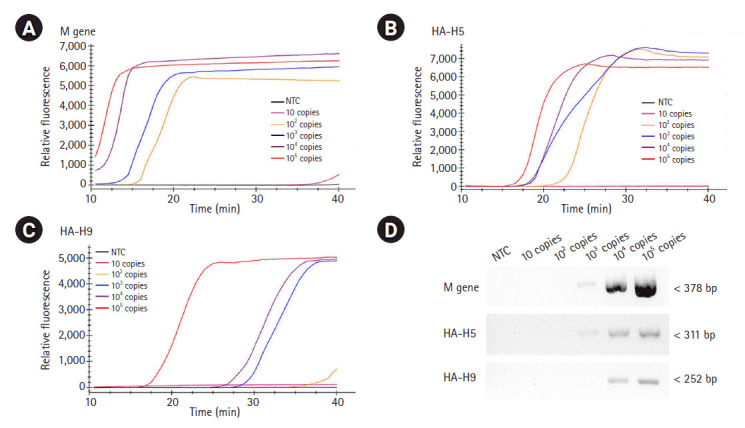
Sensitivity of RT-LAMP and RT-PCR. The template RNAs, M (A), H5-HA (B), and H9-HA (C), were 10-fold serially diluted (ranging from 10 to 10^5^ RNA copies) and applied for each real-time RT-LAMP assay. The x-axis represents the time for the RT-LAMP reaction; the y-axis represents the relative fluorescent signal. (D) The same template RNAs were applied for conventional RT-PCR using a certified commercial AIV detection kit (AIV Multi-tube RT-PCR Kit, iNtRON Biotechnology) and the products were electrophoresed with 1.5% agarose gel. RT-LAMP, reverse transcription loop-mediated isothermal amplification; RT-PCR, reverse transcription polymerase chain reaction; M, matrix; HA, hemagglutinin; AIV, avian influenza virus; NTC, negative control.

To evaluate the sensitivity of the assays, we examined the detection limits of the three RT-LAMP assays. To do so, the template RNAs were 10-fold serially diluted (ranging from 10 to 10^5^ RNA copies) and applied for each real-time RT-LAMP assay. The detection limit of the M gene and the H5-HA RT-LAMP assays was found to be 100 copies/reaction, while the detection limit of the H9-HA RT-LAMP assay was 1,000 copies/reaction (Fig. 4A‒4C). For objective validation of the sensitivity of our RT-LAMP assay, we performed conventional RT-PCR with a certified commercial AIV detection kit (AIV Multi-tube RT-PCR Kit, iNtRON Biotechnology) using the same template RNAs used for the RT-LAMP assays and compared the results. Our RT-LAMP assay was 10 times more sensitive than the reference RT-PCR kit. The detection limit of the RT-PCR assays for the M and H5-HA genes was 1,000 copies/reaction, and that for the H9-HA gene was 10,000 copies/reaction (Fig. 4D). Regarding the reaction time, the fluorescence signal of target-specific amplification appeared within 20 min in all three RT-LAMP assays when 10^5^ RNA copies were used. In the RT-LAMP assay for the M gene, even with 100 copies of the RNA template (the limit of detection), the amplification signal appeared within 20 min.

## Discussion

Recently emerging viral infectious diseases, including AIV, are increasing, posing a major threat to both public health and poultry farming. POCT is a new concept of laboratory testing that enables testing to be performed where an infection occurs without transporting the samples to central clinical laboratories [7]. LAMP technology is ideal for POCT due to its ease of performance without the need for sophisticated equipment or experts to operate it [13]. In particular, RT-LAMP can be useful to identify RNA viruses at remote locations where laboratory equipment is limited [12,13]. In this study, we aimed to developed rapid and highly sensitive assays for identifying AIVs through POCT in the context of monitoring AIV epidemic outbreaks. Using LAMP technology, we developed RT-LAMP assays that can detect the presence of AIV and distinguish the H5 and H9 subtypes within 20-30 minutes. Furthermore, all three RT-LAMP assays (M, H5, and H9) were approximately 10-fold more sensitive than the approved reference RT-PCR assay.

We first designed an AIV universal RT-LAMP assay by targeting the M gene, which has a common sequence across AIV subtypes. Therefore, a positive M RT-LAMP assay result indicated the presence of AIV in the sample. We also designed RT-LAMP primer sets specifically targeting H5-HA and H9-HA, as these are typical subtypes of HPAIVs and LPAIVs, respectively. All RT-LAMP primers were designed within a conserved region of AIVs isolated from diverse poultry across the world, suggesting that our RT-LAMP assays can be suitable for determining the presence of H5-HA and H9-HA regardless of the poultry or location. After designing the RT-LAMP primers, we used *in vitro*–transcribed full-length RNA fragments of the M, H5-HA, and H9-HA genes for experimental validation, because field-isolated AIV samples were not available due to their high pathogenicity.

Due to the very high level of amplification efficiency of the LAMP, false detection is a major concern with this technology. In this study, we used additives (NMF and IBA) to minimize nonspecific amplification [23] and checked the real-time amplification signal by adding a fluorescent reagent. Finally, we selected 0.4 M NMF + IBA because this condition showed an efficient target-specific amplification signal without any nonspecific amplification signal. Moreover, none of the three RT-LAMP assays showed any nonspecific reactions with non-matched template or negative controls. These results suggest that our RT-LAMP assays are specific enough to identify AIV subtypes.

When we checked the sensitivity, the detection limits of our RT-LAMP assays were 100 to 1,000 RNA copies per reaction, which were 10 times more sensitive than the approved reference RT-PCR assays (1000 to 10,000 RNA copies per reaction). These results are consistent with previous studies reporting that LAMP assays had higher sensitivity than PCR-based assays [18,19]. In particular, the reaction time of our RT-LAMP assay was less than 30 minutes, which was approximately four times quicker than that of the conventional RT-PCR assay. These results suggest that our RT-LAMP assays can identify AIV subtypes 10 times more sensitively and four times more quickly than the conventional RT-PCR assays.

In summary, the RT-LAMP assays targeting the M, H5-HA, and H9-HA genes developed in this study demonstrated a high level of sensitivity and specificity. The assays could successfully detect the existence of AIV and discriminate between the H5 and H9 subtypes with higher sensitivity and less time than the conventional RT-PCR assays. This method could be a useful POCT tool for the rapid identification of AIV infections in the field.

## Figures and Tables

**Table 1.** Primer sets designed for the detection of the M gene and H5- and H9-HA genes of AIVs

Gene	Primer	Length (bp)	Sequence (5'-3')
M	F3	19	GCATCGGTCTCACAGACAG
	B3	19	ACTGGAGCTAGGGTGAGTT
	FIP^a^	46	CAGCCATCTGCTCCATAGCCTTTTTTCCACCAACCCACTAATCAGG
	BIP^a^	42	CAGCGGAAGCCATGGAGGTTTTTTCCAATGGTCCTCATCGCC
	LPF	19	GGCCAGCACCATTCTGTTT
	LPB	18	AGGCTAGGCAGATGGTGC
H5-HA	F3	23	GCATACAAAATTGTCAAGAAAGG
	B3	19	ACTATTTCTGAGTCCAGTC
	FIP^a^	52	CGCMCCTATTGGAGTTTGACATTTTTTCTCAACAATTATGAAAAGTGA
	BIP^a^	52	TCTAGYATGCCRTTCCACAATATACTTTTGCAAGGACTAATTTGTTTGATTT
	LF	20	GTGTTGCAGTGGCCATACTC
	LB	21	ACCCTCTCACCATCGGGGAAT
H9-HA	F3	19	GAGAATCCTGAAGACCGAT
	B3	19	CCCTCTACCTGATGTAGCA
	FIP^a^	48	GTGGAATGGCAATGTCGTATTCAAATTTTTTAAATAGTGGCAACTGCG
	BIP^a^	46	AGCAAGTATGCATTTGGGAACTTTTTGCACATTTCTTAGACCAACT
	LF	20	CCTTTCTCAGTTTGGCACTG
	LB	25	GTTGGAGTGAAGAGTCTCAAACTGG

M, matrix; HA, hemagglutinin; AIV, avian influenza virus; RT-LAMP, reverse transcription loop-mediated isothermal amplification.

a Each inner primer (FIP and BIP) of RT-LAMP had two binding regions (F1c + F2 and B1c + B2, respectively) connected by a TTTT spacer.

## Supplementary Materials

Supplementary Figure 1. Optimization of RT-LAMP assay. Two to three different primer sets for each target gene (M, H5-HA and H9-HA) were applied for RT-LAMP assay and the products were electrophoresed with 1.5% agarose gel. The primer set demonstrated good target specific amplification and no non-specific signal was selected (*).
