# Supplementary material for: Development of reverse transcription loop-mediated isothermal amplification assays for point-of-care testing of avian influenza virus subtype H5 and H9
Source: Genomics Inform. 2020 Dec 14;18(4):e40. doi: 10.5808/GI.2020.18.4.e40 (PMC7808867; doi:10.5808/GI.2020.18.4.e40)
Supplement: Supplementary Figure 1. — Optimization of RT-LAMP assay. Two to three different primer sets for each target gene (M, H5-HA and H9-HA) were applied for RT-LAMP assay and the products were electrophoresed with 1.5% agarose gel. The primer set demonstrated good target specific amplification and no non-specific signal was selected (*). [file gi-2020-18-4-e40-suppl.pdf]

Supplementary Data

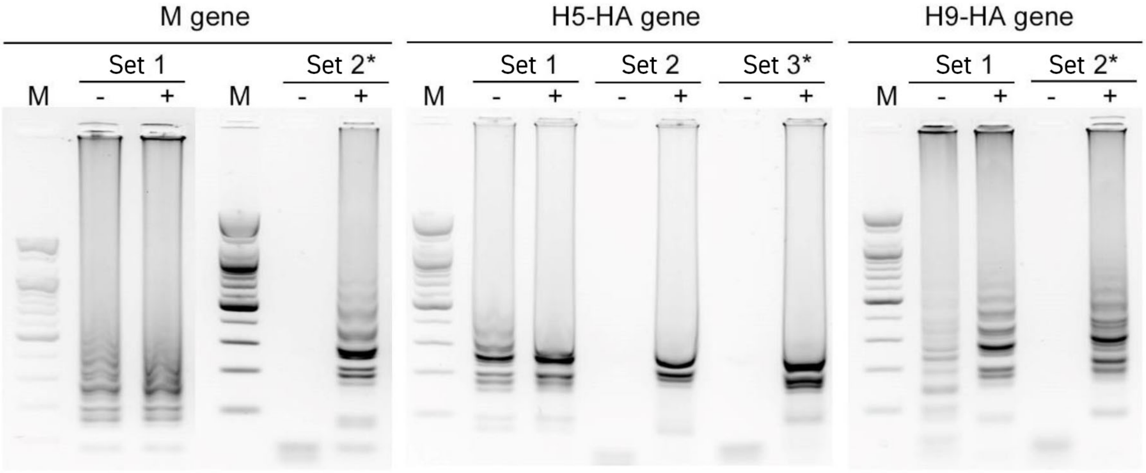

Supplementary Figure 1. Optimization of RT-LAMP assay. Two to three different primer sets for each target gene (M, H5-HA and H9-HA) were applied for RT-LAMP assay and the products were electrophoresed with 1.5% agarose gel. The primer set demonstrated good target specific amplification and no non-specific signal was selected (*).
